# Novel Gene Polymorphisms for Stable Warfarin Dose in a Korean Population: Genome-Wide Association Study

**DOI:** 10.3390/biomedicines11082308

**Published:** 2023-08-19

**Authors:** Jung Sun Kim, Sak Lee, Jeong Yee, Kyemyung Park, Eun Jeong Jang, Byung Chul Chang, Hye Sun Gwak

**Affiliations:** 1 Graduate School of Pharmaceutical Sciences, College of Pharmacy, Ewha Womans University, Seoul 03760, Republic of Korea; junction717@ewhain.net (J.S.K.); jjjhello1@naver.com (J.Y.); heimdall01@hanmail.net (E.J.J.); 2 Department of Thoracic and Cardiovascular Surgery, Cardiovascular Research Institute, Yonsei University College of Medicine, Seoul 03722, Republic of Korea; sak911@yuhs.ac; 3 Department of Biomedical Engineering, College of Information and Biotechnology, Ulsan National Institute of Science and Technology, Ulsan 44919, Republic of Korea; kyemyung.park@unist.ac.kr; 4 Department of Thoracic and Cardiovascular Surgery, Bundang CHA Medical Center, CHA University, Seongnam 13496, Republic of Korea

**Keywords:** warfarin, *FRAS1*, *FAM201A*, *NKX2-6*, polymorphism, GWAS

## Abstract

Warfarin has a narrow therapeutic window and high intra- and inter-individual variability. Considering that many published papers on genotype-guided dosing are derived from European populations, the aim of this study was to investigate novel genetic variants associated with the variability of stable warfarin dose in the Korean population with cardiac valve replacement, using the GWAS approach. This retrospective cohort study was performed from January 1982 to December 2020 at the Severance Cardiovascular Hospital of Yonsei University College of Medicine. GWAS was performed with a lenient threshold of 5 × 10^−7^ to identify associations between genotypes and the warfarin maintenance dose, by comparing the allele frequency of genetic variants between individuals. Then, the extent of genetic and non-genetic factors on the dose variability was determined by multivariable regression analysis. The study enrolled 214 participants, and the most robust signal cluster was detected on chromosome 16 around *VKORC1.* Followed by *VKORC1*, three novel variants (*NKX2-6* rs310279, *FRAS1* rs4386623, and *FAM201A* rs1890109) showed an association with stable warfarin dose requirement in univariate analysis. The algorithm was constructed by using multivariable analysis that includes genetic and non-genetic factors, and it could explain 58.5% of the variations in stable warfarin doses. In this variability, *VKORC1* rs9934438 and *FRAS1* rs4386623 accounted for 33.0% and 9.9%, respectively. This GWAS analysis identified the fact that three novel variants (*NKX2-6* rs310279, *FRAS1* rs4386623, and *FAM201A* rs1890109) were associated with stable warfarin doses. Additional research is necessary to validate the results and establish personalized treatment strategies for the Korean population.

## 1. Introduction

Despite being approved over 60 years ago, warfarin remains a fundamental anticoagulation therapy for preventing and treating a broad range of thromboembolic disorders [1,2]. Although newer agents such as direct oral anticoagulants are available, warfarin remains the drug of choice for patients who have undergone mechanical heart valve replacement [3]. However, warfarin therapy requires frequent monitoring of the international normalized ratio (INR) to ensure its pharmacological effectiveness and to avoid complications arising from its narrow therapeutic index.

The intra- and inter-individual variability in warfarin dose requirements poses a significant challenge to maintaining optimal INR. Factors contributing to this variability include age, vitamin K intake, concomitant medications, acute and chronic disease status, ethnicity, and genetics [4,5,6]. Warfarin exerts its anticoagulant effects by interfering with the cyclic inter-conversion of vitamin K and vitamin K epoxide, resulting in the inactivation of various coagulation factors (Factor II, VII, IX, and X), as well as the anticoagulant proteins C, S, and Z [7]. It is a racemic mixture of R and S enantiomers. The S enantiomer, which is 3–5 times more potent than the R enantiomer, is metabolized primarily by the cytochrome P450 (CYP) 2C9, while the R form undergoes metabolism through CYP1A2 and CYP3A4 [8]. Consequently, genetic variations in the genes encoding vitamin K epoxide reductase complex subunit 1 (*VKORC1*) and *CYP2C9* have been widely acknowledged as major contributors to inter-individual differences in stable warfarin doses [9].

Distinct variations in minor allele frequency (MAF) among ethnic groups result in significant disparities in warfarin dosage requirements [10]. For example, the variant allele frequency of *VKORC1* rs9923231 C>T is 88% in Asians, compared to only 5% in individuals of African ancestry [11,12]. The CYP2C9*2 (rs1799853) and CYP2C9*3 (rs1057910) variants are more prevalent in those with European ancestry (MAF 10% and 7%, respectively) than in Asians (MAF < 1% and 3%, respectively). Given that much of the existing literature on genotype-guided dosing is derived from European populations, it is crucial to develop Asian-specific algorithms for individualized therapy [10]. 

Genome-wide association studies (GWASs) provide a useful approach for identifying potential candidate genes related to population-specific variants. This method involves a comprehensive analysis of genetic variations across the entire genome to establish potential connections between genotype and phenotype [13]. Several studies have employed GWAS to propose novel genetic polymorphisms that may be associated with variations in warfarin maintenance doses among different populations [14,15,16]. However, there is a scarcity of research focusing on Asian-specific variants in the context of warfarin dose requirements, and to date, no GWAS has been conducted specifically within the Korean population. Consequently, this study aims to investigate novel genetic variants associated with the variability of stable warfarin doses in Korean individuals who have undergone cardiac valve replacement using the GWAS approach.

## 2. Materials and Methods

### 2.1. Study Participants and Data Collection

We conducted a retrospective cohort study using prospectively collected data from January 1982 to December 2020. The study population consisted of individuals aged 18 years or older who underwent mechanical heart valve replacement and subsequently received warfarin treatment at the Severance Cardiovascular Hospital of Yonsei University College of Medicine. The primary outcome was defined as the mean stable dose (mg/day), representing the average warfarin dosage necessary to achieve an INR of 2.0 to 3.0 for a minimum of three consecutive measurements.

Clinical data were obtained by reviewing electronic or scanned medical records, which included demographic information (age, sex, body weight, height, and body mass index), valve position, comorbidities, co-medications, INR values, and stable warfarin doses. All participants were required to provide written informed consent before participating in the study. The study protocol was reviewed and approved by the Institutional Review Board (IRB) of Yonsei University Medical Center (IRB numbers: 4-2009-0283 and 4-2020-0855). This study adhered to the ethical principles outlined in the 1694 Declaration of Helsinki and its subsequent amendments. It obtained ethics committee approval to ensure compliance with the necessary ethical standards for human research.

### 2.2. Genotyping and Sample Quality Control

Genomic DNA was extracted from ethylenediaminetetraacetic acid samples using the QIAamp DNA Blood Mini Kit (QIAGEN GmbH, Hilden, Germany). The AxiomTM Asia Precision Medicine Research Array (Thermo Fisher Scientific, Waltham, MA, USA) was employed for genotyping, and the genetic testing was entrusted to DNALink^®^. Out of more than 750,000 markers included in this genotyping array, approximately 540,000 markers were from South and East Asian populations. In the assay, 200 ng of genomic DNA was employed. After amplification, the arrays were stained and visualized on a GeneTitan MC Instrument (Affymetrix, Santa Clara, CA. USA). The Affymetrix GeneChip Command Console facilitated raw data generation, while genotype calling was performed using the AxiomGT1/BRLMM-P algorithm through Affymetrix Power Tools software version 2.11.3 (Thermo Fisher Scientific, Waltham, MA, USA).

Quality control (QC) procedures encompassed assessments of sex inconsistencies, sample relatedness, call rate, and hetero rate. After dish quality check (call rate and plate quality control) had been carried out, a relationship test was performed. The GWAS analyzed single-nucleotide polymorphisms (SNPs) with a MAF above 1%, call rate > 95%, and Hardy–Weinberg equilibrium at a threshold of *p* > 10^−4^. 

### 2.3. Genome-Wide Association Studies (GWAS)

GWAS was conducted using PLINK software version 1.07 to evaluate the association between genetic variant allele frequency and stable warfarin doses (mg/day). Post-QC filtering, 525,953 SNPs were included in the analysis. We performed a linear regression analysis in PLINK software for these SNPs, using an additive genetic model. The association results were adjusted using covariates, such as age and sex. Then, GWAS results were visualized using the Manhattan plots and quantile-quantile (Q-Q) plots. 

Functional annotation and identification of likely causal variants were performed using FUMA’s SNP2GENE in conjunction with fine-mapping analysis [17]. Significant independent SNPs were identified from GWAS results and lead SNPs were selected if pairwise SNPs demonstrated R^2^ less than 0.1. To recruit enough variables, we used a relaxed threshold of 5 × 10^−7^ for SNP selection. Linkage disequilibrium (LD) blocks were merged into a genomic locus if the maximum distance between them was 250 kb. Reference data for LD analyses consisted of genetic data from East Asian populations in 1000 G phase 3.

We utilized Haploreg v4.2 to obtain information on MAF in Asian populations for the significant independent SNPs discovered [11]. Additionally, we collected details of the SNPs such as chromosomal position, reference/alternate alleles, and functional information from the SNP database of the National Center for Biotechnology Information [12].

### 2.4. Statistical Analysis

Categorical variables were analyzed with Fisher’s exact test or the chi-square test. The independent samples *t*-test was used to compare continuous variables between two groups while one-way ANOVA followed by post hoc Tukey’s b test were used for multiple comparisons among more than two different genotype groups. Continuous variables were presented as mean ± standard deviation (SD), with a *p*-value < 0.05 considered statistically significant. For the analysis, both dominant and recessive models were applied, and the most suitable model was selected by considering both effect size and statistical significance. 

To analyze the contribution of associated SNPs to warfarin dose variability, univariate and multivariable analyses were conducted. For multivariable linear regression analysis, variables with *p* value less than 0.05 in the univariate analysis, in addition to age at operation and sex, were included. The stepwise selection was employed to select the best model, entering variables with *p* < 0.05 and removing them when *p* > 0.10. All statistical analyses were performed using IBM SPSS Statistics, version 20 software (International Business Machines Corp., New York, NY, USA).

## 3. Results

A total of 229 patients who underwent heart valve replacement therapy during the study period were assessed, and 214 patients with stable warfarin doses were included in the analysis. The average age of participants was 58.3 ± 10.1 years, with 143 females (66.8%) represented in the cohort (Table 1). The median follow-up duration was 14.2 years (range: 1.0–29.7 years), and each patient had an average of 26.2 INR measurements. The mean stable warfarin dose required to maintain the target INR for three consecutive measurements was 5.5 ± 1.9 mg/day, with substantial inter-individual variability ranging from 2.0 to 14.1 mg/day. Patients receiving angiotensin receptor blockers (ARBs) and diuretics required significantly lower doses compared to those not on these co-medications (*p* = 0.009 and 0.001, respectively).

**Figure 1:**
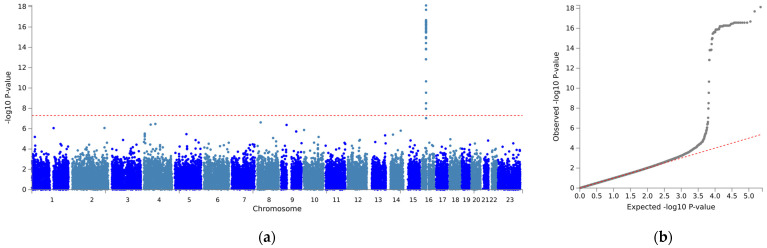
Manhattan plot of a genome-wide association for mean stable dose. (**a**) Manhattan plot (**b**) quantile-quantile (Q-Q) plot. (**a**) The vertical axis indicates the value of –log(*p*-value) for genome-wide association analysis, and the horizontal axis indicates chromosome number. The red line indicates the genome-wide significance level (*p* < 5 × 10^−8^).

**Figure 2:**
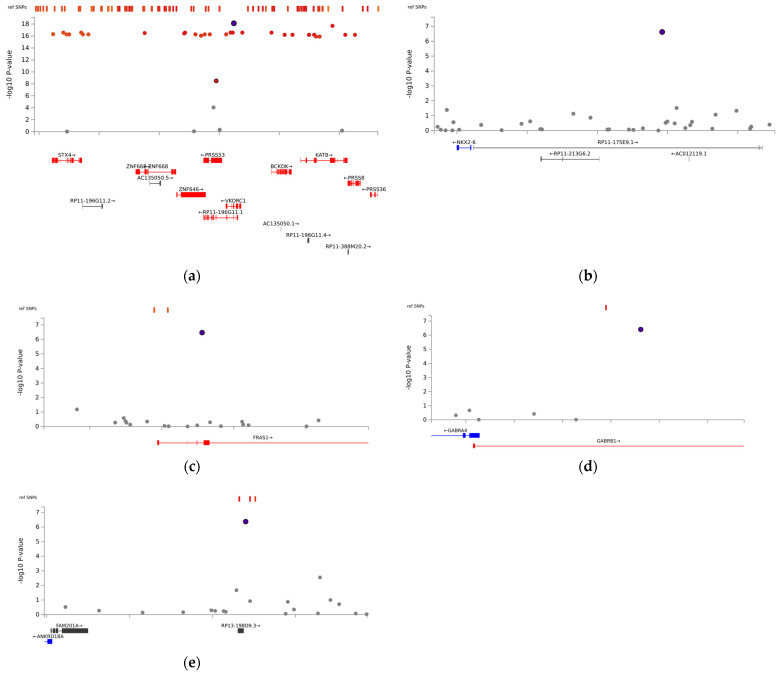
Locus-specific plots of lead single-nucleotide polymorphisms (SNPs): (**a**) *VKORC1* rs9934438, (**b**) *NKX2-6* rs310279, (**c**) *FRAS1* rs4386623, (**d**) *GABRB1* rs117496075, and (**e**) *FAM201A* rs1890109. The purple circle represents top lead SNPs, while red circles indicate independent significant SNPs.

The association analysis between mean stable warfarin doses and SNPs is illustrated in Figure 1. With a relaxed genome-wide significance threshold of 5 × 10^−7^, fine-mapping analysis to pinpoint causal variants revealed 13 independent SNPs (Supplementary Table S1) and five lead SNPs: *VKORC1* rs9934438, Fraser extracellular matrix complex subunit 1 (*FRAS1*) rs4386623, family with sequence similarity 201 member A (*FAM201A*) rs1890109, NK2 Homeobox-6 (*NKX2-6*) rs310279, and gamma-aminobutyric acid type A receptor subunit beta1 (*GABRB1*) rs117496075 (Figure 2). The most pronounced signal cluster was identified on chromosome 16 surrounding *VKORC1*, with *VKORC1* rs9934438 exhibiting the lowest univariate *p*-value of 8.41 × 10^−20^. Apart from this SNP, none of the SNPs achieved a genome-wide significance of 5 × 10^−8^. 

The relationship between stable warfarin dose and genotypes was examined using univariate analysis (Table 2). All lead SNPs, except for *GABRB1* rs17496075, demonstrated a significant difference in mean stable warfarin doses. Patients possessing the *VKORC1* rs9934438 GG genotype required 9.74 ± 2.18 mg/day, whereas those with AA and AG genotypes needed 5.41 ± 1.84 mg/day (*p* = 6.23 × 10^−6^). Notably, *FRAS1* rs4386623 A allele carriers necessitated significantly higher warfarin doses compared to non-carriers (9.70 ± 2.79 vs. 5.39 ± 1.80 mg/day, *p* = 4.45 × 10^−7^). 

A stepwise linear regression analysis was conducted to ascertain the relative contributions of independent variables to the variability of stable warfarin doses (Table 3). Model I, which incorporated age at operation, sex, and factors with *p* < 0.05 from the univariate analysis, accounted for 53.1% of the variability in stable warfarin dose. Genetic and non-genetic factors (age at operation, diuretics, and ARB) explained 48.2% and 6.4% of warfarin dose variability, respectively. The *VKORC1* rs9934438 allelic variation was the most influential, explaining 33.0% of dose variability, followed by *FRAS1* rs4386623 at 9.9% and *FAM201A* rs1890109 at 4.0%.

Since CYP2C9*3 is a well-established genetic factor in warfarin treatment, we developed Model II, which included this SNP, and performed additional analysis. Model II, incorporating CYP2C9*3, predicted further dose variability (R^2^ 0.585), with this SNP contributing to 5.2% of the variability.

## 4. Discussion

In this GWAS analysis, we identified three novel variants (*FRAS1* rs4386623, *FAM201A* rs1890109, and *NKX2-6* rs310279) that were significantly associated with stable warfarin dose requirements in patients who underwent heart valve replacements. Notably, patients with polymorphisms in these SNPs necessitated higher stable doses of warfarin. Additionally, the concurrent use of diuretics and ARBs emerged as significant factors contributing to an increase in stable warfarin doses. Our multivariable analysis, encompassing both genetic and non-genetic factors, accounted for over 50% of the variation in stable warfarin dosing. 

Among the identified variants, *FRAS1* rs4386623 explained 9.9% of the variability, making it the second-strongest variable after *VKORC1* in our study. Mutations in *FRAS1* are known to be associated with Fraser syndrome, a disorder characterized by multisystem abnormalities, including malformations in the craniofacial, urogenital, and respiratory systems [18]. While numerous studies have examined the involvement of *FRAS1* mutation in the pathogenesis and prognosis of diseases, there is limited research exploring its association with drug-related variations. A GWAS study conducted on sub-Saharan black African patients revealed that *FRAS1* rs7676083 was associated with a significant difference in the ratio of warfarin enantiomers and metabolites (RS-10hydroxywarfarin/RS-warfarin) [19]. From a GWAS study of 74 invasive epithelial ovarian cancer patients observed at the Mayo Clinic, it was found that some genes including *FRAS1* may explain inter-patient variation in clinical response to platinum–taxane therapies [20]. Additionally, the result of whole-exome sequencing and the GWAS study demonstrated that *FRAS1* polymorphism had significant influences on the pharmacokinetic parameters of dabigatran, a direct oral anticoagulant in a healthy Chinese population [21]. 

The rs4386623 of *FRAS1* is an intron, but intron regions may impact mRNA splicing, thereby modifying protein expression or activity [22,23]. This SNP is located at the 4q21.21 locus, and this region has been associated with the 4q deletion syndrome, which is a genetic disorder characterized by interstitial or terminal deletions on the long arm of chromosome 4 [24]. Affected individuals typically exhibit craniofacial abnormalities and delayed growth, and may also present congenital heart defects such as atrial septal defect (ASD), ventricular septal defect (VSD), and patent ductus arteriosus [24,25]. In a case report by Xu et al., the critical region for cardiovascular involvement in 4q deletion syndrome was narrowed down to the chromosomal region between 4q32.2 and q34.3. However, another study conducted in China reported that chromosome 4q21.1q21.21 (position: 78,328,101–79,689,207) was identified as having an uncertain but significant association with ASD and VSD in chromosomal microarray analysis [26,27]. These congenital heart diseases can disrupt blood flow to the lungs, resulting in an increased risk of serious complications such as arrhythmia, high blood pressure, and stroke [28,29]. However, the biological mechanism underlying the observed correlation between warfarin doses and *FRAS1* rs4386623 needs to be elucidated in further research.

*FAM201A* rs1890109, another novel variant, accounted for 4.7% of warfarin dose variability in Model II. FAM201A, a family of long non-coding RNA, was previously demonstrated to be implicated in various diseases, particularly cancers [30,31]. FAM201A participated in cell proliferation, migration, and invasion by regulating ATP-binding cassette transporter E1. Consequently, the upregulation influenced the survival of the patients with lung squamous cell cancer [30,31]. Aside from its role in cancers, a decrease in FAM201A expression has been linked to osteonecrosis of the femoral head [32]. Recently, Chen et al. revealed that FAM201A was associated with an increased susceptibility to atrial fibrillation through ceRNA network analysis [33]. This study indicated that the down-regulation of FAM201A could potentially be used as a predictive marker for atrial fibrillation susceptibility. According to the GTEx portal, eQTL analysis revealed that this SNP was recorded as a significant expression quantitative trait locus for the FAM201A (*p* = 2.3 × 10^−6^) in the left ventricle [34]. The variant allele of rs1890109 is related to a decreased expression of FAM201A. Considering that *FAM201A* polymorphism is associated with atrial fibrillation, the effects of rs1890109 variant on gene expression may increase the risk of thrombosis and interfere with the coagulation cascade, consequently altering stable warfarin dose requirements. While this may provide preliminary insight into how FAM201A impacts warfarin metabolism, the detailed mechanism must be further explored.

The association between *NKX2-6* and stable warfarin doses was observed in our study, accounting for 2.0% of the variability. NKX2-6, a critical transcription factor, is involved in the development of the heart’s dorsal vessel. Mutations in this gene and its counterpart, NKX*2-5*, have been linked to various congenital heart defects [35]. During embryogenesis in mice, the expression of NKX*2-6* occurs at the opposite poles of the developing heart [36]. While the disruption of NKX*2-6* did not result in apparent heart malformations, the expansion of NKX*2-5* mRNA expression into these regions, where NKX*2-6* was typically present, was observed. This indicates that functional compensation occurred for the loss of NKX*2-6*. Furthermore, double knockout mouse embryos have demonstrated overlapping functions of NKX*2-6* and NKX*2-5*, as the development of the atria was less advanced in these embryos. This provides further evidence of the essential role of these two genes in cardiac development [37]. Some research has proposed that genetic variations in these transcription factors were related to the higher risk of atrial fibrillation, suggesting that *NKX2-6* mutations might also increase thromboembolism risk [38,39]. Hence, the observed influence of *NKX2-6* genetic variations on stable warfarin dose requirements in our study could be attributed to alterations in the coagulation system, derived from abnormal cardiovascular function. 

Our findings indicated that co-administration of diuretics significantly affected stable warfarin doses. Diuretics are frequently prescribed to relieve pulmonary congestion [40]. In our previous research, we demonstrated that the concurrent use of warfarin and diuretics may enhance warfarin’s anticoagulant effect in Korean patients with atrial fibrillation [41]. There was a notable difference in stable warfarin between individuals who used hydrochlorothiazide and those who did not (2.97 ± 1.10 mg/day vs. 3.58 ± 1.14 mg/day, *p* = 0.009). However, when the specific subtype of diuretics was not considered, the significance became marginal (*p* = 0.069). On the contrary, a retrospective study conducted in two hospitals in the United States suggested that diuretics (namely furosemide and hydrochlorothiazide) did not significantly alter the INR in patients on stable warfarin therapy [42]. It is important to note that the majority of participants in this study were Caucasians (88.6%), without any Asians included. Therefore, this difference in ethnic composition could potentially explain the inconsistent findings, as there may be variations due to ethnic diversity or possible interaction between genetic and environmental factors.

In our study, the concurrent use of ARBs decreased the required warfarin dose compared to that for non-users. An in vitro study investigating the effects of ARBs on the CYP2C9 activity in human liver microsomes has been conducted [43]. The finding of this study indicated that specific chemical structures of ARBs significantly affected their affinity to the CYP2C9 enzyme, exerting inhibitory effects on the enzyme. However, a 30-day open-label, single-period study reported that telmisartan treatment reduced mean warfarin trough plasma concentration but did not change the INR [44]. Therefore, the impact of ARBs on the requirements of stable warfarin doses remains unclear, and requires further investigation.

Previous GWAS findings revealed that *VKORC1* and *CYP2C9* significantly influenced the inter-variability in warfarin dose requirement [16,45,46,47]. In populations of European descent, *VKORC1* and *CYP2C9* accounted for 25% and 9% of the dose variance, respectively [46]. Our study results indicated that *VKORC1* and *CYP2C9* contributed 33.0% and 5.2% of the variance, respectively. However, *CYP2C9* did not achieve a genome-wide significant *p*-value in our GWAS, which could be due to allele frequency variations across different populations [10]. This result is in accordance with the findings in a GWAS conducted with Japanese patients [48]. In that study, only SNPs located near the *VKORC1* gene demonstrated a significant association with therapeutic warfarin dose at a genome-wide level. Neither SNPs in the *CYP2C9* gene nor the *CYP4F2* gene exhibited a genome-wide significant association. The genetic variation observed across different populations highlights the importance of employing an algorithm specifically designed for the Asian population when predicting INR levels.

Despite its retrospective design, our study represents the first GWAS to explore novel candidate genes related to stable warfarin dose requirement in a Korean population. We identified several novel variants significantly impacting warfarin dose requirement. Furthermore, our study exclusively included patients with heart valve replacement, ensuring homogeneity in patient characteristics and target INR. However, as this investigation was conducted in a single center with a limited sample size, the results might not be generalizable to populations with diverse genetic backgrounds. Also, a less-strict genome-wide threshold of 5 × 10^−7^ was used to recruit more variables in the study. As determining the correct *p*-value for statistical significance is crucial for controlling the number of false-positive associations, additional research is required to validate our algorithm in a replication cohort to confirm its efficacy.

## 5. Conclusions

Our GWAS has uncovered three previously unreported genetic variants (*FRAS1* rs4386623, *FAM201A* rs1890109, and *NKX2-6* rs310279) that demonstrate associations at a relaxed significance threshold with stable warfarin dose requirements in heart valve replacement patients. Further investigation is necessary to validate these findings and develop individualized treatment approaches tailored specifically to the Korean population.

## Figures and Tables

**Table 1:** Demographic characteristics and warfarin stable dose (mg/day).

Characteristics	Number (%) (*n* = 214)	Stable Dose (mg/day) (mean ± SD)	*p*-Value
Age (year)	58.3 ± 10.1 ^g^		
Age at operation (year)	43.5 ± 11.2 ^g^		
Sex			0.217
Male	71 (33.2)	5.72 ± 1.95	
Female	143 (66.8)	5.38 ± 1.92	
Body mass index (kg/m^2^)			0.480
<25	160 (74.8)	5.44 ± 1.83	
≥25	54 (25.2)	5.65 ± 2.23	
Valve position			0.876
Aortic	47 (22.0)	5.68 ± 1.60	
Mitral	106 (49.5)	5.46 ± 2.03	
Double ^a^	41 (19.2)	5.38 ± 2.17	
Tricuspid ^b^	16 (7.5)	5.34 ± 1.77	
Comorbidity			
Hypertension			0.174
Yes	18 (8.4)	5.18 ± 1.70	
No	196 (91.6)	5.59 ± 2.00	
Diabetes mellitus			0.940
Yes	18 (8.4)	5.49 ± 1.94	
No	196 (91.6)	5.49 ± 1.94	
Congestive heart failure			0.717
Yes	47 (22.0)	5.40 ± 2.16	
No	167 (78.0)	5.52 ± 1.87	
Atrial fibrillation			0.095
Yes	124 (57.9)	5.30 ± 1.84	
No	90 (42.1)	5.75 ± 2.03	
Rheumatic disease			0.650
Yes	122 (57.0)	5.44 ± 1.90	
No	92 (43.0)	5.56 ± 1.99	
Myocardial infarction			0.825
Yes	4 (1.90)	5.70 ± 2.01	
No	210 (98.1)	5.49 ± 1.94	
Comedication			
Antiplatelet drugs ^c^			0.582
Yes	8 (4.1)	5.15 ± 1.10	
No	188 (95.9)	5.53 ± 1.95	
INR-increasing drugs ^d^			0.359
Yes	1 (0.5)	3.75	
No	198 (99.5)	5.52 ± 1.92	
INR-decreasing drugs ^e^			0.353
Yes	2 (1.0)	6.77 ± 2.50	
No	197 (99.0)	5.50 ± 1.91	
ARBs			0.009
Yes	46 (21.9)	4.81 ± 1.51	
No	164 (78.1)	5.66 ± 2.00	
ACEIs			0.405
Yes	32 (15.2)	5.21 ± 1.88	
No	178 (84.8)	5.52 ± 1.94	
Diuretics ^f^			0.001
Yes	90 (42.9)	4.98 ± 1.89	
No	120 (57.1)	5.84 ± 1.89	
Calcium channel blockers			0.389
Yes	31 (15.5)	5.78 ± 2.01	
No	169 (84.5)	5.46 ± 1.90	
Statins			0.455
Yes	8 (4.0)	5.02 ± 1.43	
No	192 (96.0)	5.53 ± 1.93	

ACEIs: angiotensin-converting-enzyme inhibitors, ARBs: angiotensin receptor blockers, INR: international normalized ratio, SD: standard deviation. ^a^ Aortic plus mitral valve. ^b^ Any valve replacements including tricuspid valve. ^c^ including aspirin, cilostazol, and clopidogrel. ^d^ including amiodarone and fluconazole. ^e^ including carbamazepine, phenytoin, and rifampin. ^f^ including amiloride, furosemide, hydrochlorothiazide, spironolactone, and torsemide. ^g^ Mean ± SD.

**Table 2:** Effects of lead single-nucleotide polymorphisms on warfarin stable dose.

CHR	Gene Polymorphism	Position	Allele Frequency	Grouped Genotypes	Number (%)	Stable Dose (mg/day) (mean ± SD)	*p*-Value
16	*VKORC1* rs9934438	31104878	G:A = 0.11:0.89	GG	4 (3.3)	9.74 ± 2.18	6.23 × 10^−6^
				AG, AA	209 (96.7)	5.41 ± 1.84	
4	*FRAS1* rs4386623	78991190	G:A = 0.99:0.01	GG	209 (95.9)	5.39 ± 1.80	4.45 × 10^−7^
				GA, AA	5 (4.1)	9.70 ± 2.79	
9	*FAM201A* rs1890109	38643996	A:G = 0.98:0.02	AA	206 (94.6)	5.39 ± 1.75	0.029
				AG, GG	7 (5.4)	9.04 ± 3.39	
8	*NKX2-6* rs310279	23618463	A:G = 0.98:0.02	AA	205 (94.0)	5.36 ± 1.78	9.30 × 10^−3^
				AG, GG	8 (6.0)	8.84 ± 2.78	
4	*GABRB1* rs117496075	47013091	G:A = 0.98:0.02	GG	208 (95.4)	5.39 ± 1.78	0.055
				GA, AA	6 (4.6)	8.99 ± 3.54	

CHR: chromosome, SD: standard deviation.

**Table 3:** Multivariable linear regression analysis to identify single-nucleotide polymorphisms with warfarin stable dose.

Predictors	Model I			Model II		
	β (95% CI)	R^2^	*p*-Value	β (95% CI)	R^2^	*p*-Value
Age at operation (year)	−0.02 (−0.04, −0.01)	0.011	0.027	−0.02 (−0.04, −0.01)	0.030	0.013
ARBs	−0.54 (−1.00, −0.09)	0.021	0.019	−0.56 (−0.99, −0.14)	0.015	0.010
Diuretics	−0.46 (−0.85, −0.06)	0.032	0.024	−0.39 (−0.76, −0.02)	0.009	0.040
*VKORC1* rs9934438 per A allele	−2.04 (−2.44, −1.64)	0.330	<2.0 × 10^−16^	−1.98 (−2.44, −1.64)	0.330	<2.0 × 10^−16^
*FRAS1* rs4386623 (0 = GG, 1 = GA,AA)	3.25 (2.04, 4.45)	0.099	3.07 × 10^−7^	3.10 (2.04, 4.45)	0.099	1.91 × 10^−7^
*FAM201A* rs1890109 (0 = AA, 1 = AG,GG)	1.71 (0.65, 2.76)	0.040	0.002	1.91 (0.91, 2.90)	0.047	2.00 × 10^−4^
*NKX2-6* rs310279 (0 = AA, 1 = AG,GG)	1.43 (0.41, 2.44)	0.013	0.006	1.32 (0.36, 2.28)	0.020	0.007
*CYP2C9* rs1057910 per C allele				−1.75 (−2.41, −1.08)	0.052	6.87 × 10^−7^
Adjusted R^2^		0.531			0.585	

ARBs: angiotensin receptor blockers; CI: confidence interval; Model I included variables of sex, age at operation, diuretics, ARB, *VKORC1* rs9934438, *FRAS1* 4386623, *FAM201A* rs1890109, and *NKX2-6* rs310249. Model II included variables of sex, age at operation, diuretics, ARBs, *VKORC1* rs9934438, *FRAS1* 4386623, *FAM201A* rs1890109, *NKX2-6* rs310249, and *CYP2C9* rs1057910.

## Data Availability

Data is contained within the article. The data presented in this study is available upon reasonable request from the corresponding author.

## Supplementary Materials

The following supporting information can be downloaded at: https://www.mdpi.com/article/10.3390/biomedicines11082308/s1, Table S1: Single-nucleotide polymorphisms reached genome significance at *p*-value < 5 × 10^−7^.
