# Correction: Kim et al. Novel Gene Polymorphisms for Stable Warfarin Dose in a Korean Population: Genome-Wide Association Study. *Biomedicines* 2023, *11*, 2308

**DOI:** 10.3390/biomedicines13051238

**Published:** 2025-05-20

**Authors:** Jung Sun Kim, Sak Lee, Jeong Yee, Kyemyung Park, Eun Jeong Jang, Byung Chul Chang, Hye Sun Gwak

**Affiliations:** 1 Graduate School of Pharmaceutical Sciences, College of Pharmacy, Ewha Womans University, Seoul 03760, Republic of Korea; junction717@ewhain.net (J.S.K.); jjjhello1@naver.com (J.Y.); heimdall01@hanmail.net (E.J.J.); 2 Department of Thoracic and Cardiovascular Surgery, Cardiovascular Research Institute, Yonsei University College of Medicine, Seoul 03722, Republic of Korea; sak911@yuhs.ac; 3 Department of Biomedical Engineering, College of Information and Biotechnology, Ulsan National Institute of Science and Technology, Ulsan 44919, Republic of Korea; kyemyung.park@unist.ac.kr; 4 Department of Thoracic and Cardiovascular Surgery, Bundang CHA Medical Center, CHA University, Seongnam 13496, Republic of Korea

## Text Correction

There was an error in the original publication [1]. We applied a relaxed *p*-value threshold of 5 × 10^−7^, which was mentioned in the Results section. However, this threshold was not stated in the Abstract and Conclusions sections, which may mislead readers into assuming that the conventional genome-wide significance threshold (5 × 10^−8^) was used.

A correction has been made to the Abstract:

GWAS was performed with a lenient threshold of 5 × 10^−7^ to identify associations between genotypes and the warfarin maintenance dose, by comparing the allele frequency of genetic variants between individuals.

A correction has been made to the Conclusions:

Our GWAS has uncovered three previously unreported genetic variants (*FRAS1* rs4386623, *FAM201A* rs1890109, and *NKX2-6* rs310279) that demonstrate associations at a relaxed significance threshold with stable warfarin dose requirements in heart valve replacement patients.

The authors state that the scientific conclusions are unaffected. This correction was approved by the Academic Editor. The original publication has also been updated.
