# Supplementary material for: Novel Gene Polymorphisms for Stable Warfarin Dose in a Korean Population: Genome-Wide Association Study
Source: Biomedicines. 2023 Aug 19;11(8):2308. doi: 10.3390/biomedicines11082308 (PMC10452379; doi:10.3390/biomedicines11082308)
Supplement: Supplementary file 1 [file biomedicines-11-02308-s001.zip › biomedicines-2520037-supplementary.pdf]

Supplementary Materials

Table S1. Single nucleotide polymorphisms reached genome significance at p value < 5 × 10^{-7}.

CHR	Position	β	SE	R2	T	p	Gene	rsID
16	31104878	2.341	0.2319	0.3257	10.09	8.41×10^{-20}	*VKORC1*	rs9934438
16	30947572	2.402	0.2449	0.3131	9.806	6.05×10^{-19}	*FBXL19*	rs7200879
16	31137712	2.335	0.2384	0.3125	9.794	6.57×10^{-19}	*KAT8*	rs889548
16	31075175	2.203	0.2279	0.3079	9.665	1.62×10^{-18}	*ZNF668*	rs2303223
16	31011183	2.198	0.2291	0.3028	9.595	2.46×10^{-18}	*STX1B*	rs4889606
16	31014179	2.181	0.2283	0.301	9.555	3.23×10^{-18}	*STX1B*	rs9926533
16	31048079	2.181	0.2283	0.301	9.555	3.23×10^{-18}	*VKORC1*	rs10871454
16	31054040	2.181	0.2283	0.301	9.555	3.23×10^{-18}	*STX4*	rs2288004
16	31088347	2.181	0.2283	0.301	9.555	3.23×10^{-18}	*VKORC1*	rs749671
16	31088625	2.181	0.2283	0.301	9.555	3.23×10^{-18}	*ZNF646*	rs749670
16	31103796	2.181	0.2283	0.301	9.555	3.23×10^{-18}	*VKORC1*	rs2359612
16	31104509	2.181	0.2283	0.301	9.555	3.23×10^{-18}	*VKORC1*	rs8050894
16	31107689	2.181	0.2283	0.301	9.555	3.23×10^{-18}	*BCKDK*	rs9923231
16	31117413	2.181	0.2283	0.301	9.555	3.23×10^{-18}	*BCKDK*	rs2032915
16	31044683	2.211	0.2313	0.3042	9.559	3.44×10^{-18}	*STX4*	rs58726213
16	31037020	2.181	0.2289	0.3007	9.525	4.07×10^{-18}	*STX4*	rs11150604
16	31049155	2.176	0.2301	0.2966	9.455	6.32×10^{-18}	*STX4*	rs55979739
16	31050023	2.176	0.2301	0.2966	9.455	6.32×10^{-18}	*STX4*	---
16	31050033	2.176	0.2301	0.2966	9.455	6.32×10^{-18}	*STX4*	---
16	31056433	2.176	0.2301	0.2966	9.455	6.32×10^{-18}	*ZNF668*	rs35468353
16	31092075	2.176	0.2301	0.2966	9.455	6.32×10^{-18}	*ZNF646*	rs7196726
16	31096876	2.176	0.2301	0.2966	9.455	6.32×10^{-18}	*PRSS53*	rs4468641
16	31102321	2.176	0.2301	0.2966	9.455	6.32×10^{-18}	*VKORC1*	rs7294
16	31121793	2.15	0.2281	0.2954	9.429	7.54×10^{-18}	*BCKDK*	rs14235
16	31124407	2.15	0.2281	0.2954	9.429	7.54×10^{-18}	*KAT8*	rs749767
16	31129895	2.15	0.2281	0.2954	9.429	7.54×10^{-18}	*KAT8*	rs9925964
16	31129942	2.15	0.2281	0.2954	9.429	7.54×10^{-18}	*KAT8*	rs1978487
16	31131614	2.15	0.2281	0.2954	9.429	7.54×10^{-18}	*KAT8*	rs4527034
16	31145219	2.15	0.2281	0.2954	9.429	7.54×10^{-18}	*PRSS8*	rs12597511
16	31054607	2.173	0.2307	0.2959	9.417	8.38×10^{-18}	*ZNF668*	rs1108431
16	31095171	2.232	0.2376	0.2949	9.394	9.79×10^{-18}	*POL3S*	rs11865038
16	31093954	2.172	0.2314	0.2956	9.388	1.05×10^{-17}	*ZNF646*	rs750952
16	30918487	2.224	0.2384	0.2912	9.332	1.44×10^{-17}	*CTF1*	rs11649653
16	30948397	2.224	0.2384	0.2912	9.332	1.44×10^{-17}	*FBXL19*	rs12917722
16	31132250	2.144	0.2299	0.291	9.328	1.48×10^{-17}	*KAT8*	rs11865499
16	31133449	2.144	0.2299	0.291	9.328	1.48×10^{-17}	*KAT8*	rs1060506
16	30995669	2.25	0.2419	0.2908	9.301	1.82×10^{-17}	*SETD1A*	rs6950
16	30928970	2.22	0.2404	0.2869	9.236	2.74×10^{-17}	*FBXL19, PRSS53*	rs12924903
16	30936081	2.22	0.2404	0.2869	9.236	2.74×10^{-17}	*FBXL19*	rs35675346
16	30999142	2.22	0.2404	0.2869	9.236	2.74×10^{-17}	*HSD3B7*	rs9938550
16	31002664	2.22	0.2404	0.2869	9.236	2.74×10^{-17}	*STX1B*	rs8062719
16	30970941	2.251	0.2438	0.2879	9.236	2.80×10^{-17}	*SETD1A*	rs1870293
16	30942625	2.222	0.2411	0.2869	9.213	3.27×10^{-17}	*FBXL19, STX1B*	rs10782001
16	30982225	2.19	0.238	0.2853	9.199	3.50×10^{-17}	*SETD1A*	rs4889603
16	30862135	2.462	0.2733	0.2769	9.01	1.22×10^{-16}	*BCL7C*	rs4889614
16	30865440	2.462	0.2733	0.2769	9.01	1.22×10^{-16}	*BCL7C*	rs4889534

16	30872471	2.462	0.2733	0.2769	9.01	1.22×10^{-16}	*BCL7C*	rs11647697
16	30878863	2.462	0.2733	0.2769	9.01	1.22×10^{-16}	*BCL7C*	rs4889634
16	30881299	2.462	0.2733	0.2769	9.01	1.22×10^{-16}	*BCL7C*	rs12920259
16	30979818	2.162	0.2421	0.279	8.929	2.40×10^{-16}	*SETD1A*	---
16	30888295	2.422	0.2719	0.2722	8.905	2.44×10^{-16}	*LOC101928736*	rs67456613
16	30890538	2.422	0.2719	0.2722	8.905	2.44×10^{-16}	*LOC101928736*	rs12926295
16	30897456	2.41	0.2716	0.2716	8.87	3.13×10^{-16}	*BCL7C*	rs11150599
16	31141993	2.075	0.2351	0.2696	8.825	4.23×10^{-16}	*KAT8*	rs1549293
16	30833321	2.423	0.2781	0.2637	8.713	8.56×10^{-16}	*ZNF629*	rs8046001
16	30837596	2.423	0.2781	0.2637	8.713	8.56×10^{-16}	*ZNF629*	rs12926237
16	30895545	2.46	0.2841	0.2613	8.659	1.22×10^{-15}	*LOC101928736*	rs4889651
16	30739919	2.385	0.2801	0.2557	8.515	3.14×10^{-15}	*SRCAP*	rs2289442
16	31016970	2.114	0.2509	0.2508	8.424	5.52×10^{-15}	*STX1B*	rs729482
16	31026427	2.114	0.2509	0.2508	8.424	5.52×10^{-15}	*STX1B, STX4, ZNF668*	rs4889609
16	31026427	2.114	0.2509	0.2508	8.424	5.52×10^{-15}	*STX1B, STX4, ZNF668*	rs4889609
16	31022639	2.114	0.2517	0.2505	8.399	6.61×10^{-15}	*STX4*	rs72800847
16	30833246	2.32	0.2791	0.2458	8.313	1.12×10^{-14}	*ZNF629*	rs9319587
16	30672719	2.239	0.2697	0.2453	8.302	1.20×10^{-14}	*FBRS*	rs885107
16	30703155	2.239	0.2697	0.2453	8.302	1.20×10^{-14}	*LOC730183*	rs7187359
16	30804257	2.239	0.2697	0.2453	8.302	1.20×10^{-14}	*ZNF629*	rs12447534
16	30822409	2.239	0.2697	0.2453	8.302	1.20×10^{-14}	*ZNF629*	rs13337900
16	30823047	2.239	0.2697	0.2453	8.302	1.20×10^{-14}	*ZNF629*	rs4889490
16	30635659	2.104	0.2649	0.2294	7.944	1.13×10^{-13}	*PRR14*	rs7186852
16	30642867	2.104	0.2649	0.2294	7.944	1.13×10^{-13}	*PRR14*	rs7197475
16	31154358	1.885	0.2543	0.2058	7.412	2.93×10^{-12}	*PRSS36*	rs78924645
16	31099011	1.347	0.2113	0.1608	6.374	1.13×10^{-9}	*PRSS53*	rs11150606
16	30554159	1.417	0.2294	0.1525	6.175	3.32×10^{-9}	*ZNF747*	rs8050463
1	1.47E+08	6.396	1.037	0.152	6.165	3.51×10^{-9}	*ACP6*	rs140111718
16	31166028	1.54	0.2616	0.1411	5.887	1.53×10^{-8}	*FUS*	rs12102776
16	31004812	2.254	0.4039	0.1297	5.581	7.36×10^{-8}	*PRSS53, FBXL19*	rs12445568
18	77294324	6.967	1.291	0.1208	5.397	1.81×10^{-7}	*CTDP1*	rs190498759
8	23618463	3.477	0.6578	0.1169	5.286	3.11×10^{-7}	*NKX2-6*	rs310279
9	38643996	3.651	0.6968	0.1151	5.239	3.89×10^{-7}	*FAM201A*	rs1890109
4	78991190	4.304	0.8258	0.114	5.212	4.45×10^{-7}	*FRAS1*	rs4386623
